# Metagenome-assembled genomes provide insight into the metabolic potential during early production of Hydraulic Fracturing Test Site 2 in the Delaware Basin

**DOI:** 10.3389/fmicb.2024.1376536

**Published:** 2024-06-12

**Authors:** Brooke Stemple, Djuna Gulliver, Preom Sarkar, Kara Tinker, Kyle Bibby

**Affiliations:** ^1^Department of Civil and Environmental Engineering and Earth Sciences, University of Notre Dame, Notre Dame, IN, United States; ^2^Oak Ridge Institute for Science and Education, Oak Ridge, TN, United States; ^3^National Energy Technology Laboratory (NETL), Pittsburgh, PA, United States; ^4^Leidos Research Support Team, Pittsburgh, PA, United States

**Keywords:** produced water microbiology, hydraulic fracturing, permian basin, wolfcamp shale, natural gas, microbiome

## Abstract

Demand for natural gas continues to climb in the United States, having reached a record monthly high of 104.9 billion cubic feet per day (Bcf/d) in November 2023. Hydraulic fracturing, a technique used to extract natural gas and oil from deep underground reservoirs, involves injecting large volumes of fluid, proppant, and chemical additives into shale units. This is followed by a “shut-in” period, during which the fracture fluid remains pressurized in the well for several weeks. The microbial processes that occur within the reservoir during this shut-in period are not well understood; yet, these reactions may significantly impact the structural integrity and overall recovery of oil and gas from the well. To shed light on this critical phase, we conducted an analysis of both pre-shut-in material alongside production fluid collected throughout the initial production phase at the Hydraulic Fracturing Test Site 2 (HFTS 2) located in the prolific Wolfcamp formation within the Permian Delaware Basin of west Texas, USA. Specifically, we aimed to assess the microbial ecology and functional potential of the microbial community during this crucial time frame. Prior analysis of 16S rRNA sequencing data through the first 35 days of production revealed a strong selection for a *Clostridia* species corresponding to a significant decrease in microbial diversity. Here, we performed a metagenomic analysis of produced water sampled on Day 33 of production. This analysis yielded three high-quality metagenome-assembled genomes (MAGs), one of which was a *Clostridia* draft genome closely related to the recently classified *Petromonas tenebris*. This draft genome likely represents the dominant *Clostridia* species observed in our 16S rRNA profile. Annotation of the MAGs revealed the presence of genes involved in critical metabolic processes, including thiosulfate reduction, mixed acid fermentation, and biofilm formation. These findings suggest that this microbial community has the potential to contribute to well souring, biocorrosion, and biofouling within the reservoir. Our research provides unique insights into the early stages of production in one of the most prolific unconventional plays in the United States, with important implications for well management and energy recovery.

## Introduction

Hydraulic fracturing accounts for the majority of new oil and natural gas wells in the United States, with the U.S. Energy Information Administration projecting at least a 1 Bcf/d increase in demand for early 2024 (Short-Term Energy Outlook – U.S., n.d.). Most of these unconventional oil and gas wells are horizontally drilled. The most prolific hydrocarbon-producing shale play in the United States is the Permian Basin in west Texas, which consists of the Delaware, Midland, and Central sub-basins. The Wolfcamp Shale is an organic-rich formation deposited throughout all three sub-basins and is responsible for approximately 4 Bcf/d of natural gas, more than one-third of the total natural gas recovered from the Permian, making it the most productive shale gas-bearing formation in this region (Permian Basin Wolfcamp and Bone Spring Shale Plays Geology Review, 2019; U.S. Energy Information Administration, 2022).

The process of hydraulic fracturing involves the injection of large volumes of fluid, typically 2–4 million gallons of water per well, accompanied by proppant with chemical additives (termed fracture fluid or frac fluid) into the shale unit (Chen et al., 2014; Stringfellow et al., 2014). This is then followed by a “shut-in” period during which the fracture fluid in the well is pressurized for up to 3 weeks. Once this initial shut-in period has ended, the well begins producing, and the flow-back phase begins. The injected fluid reacts with the shale formation over time and the produced fluid composition shifts from a flowback water to an oil/gas associated fluid with high total dissolved solids termed “produced water” (Gregory et al., 2011). During this “shut-in” time, biogeochemical and microbiology changes may occur in the reservoir that will impact the produced water (Osselin et al., 2019; Gulliver et al., 2021).

Microbial growth in oil and gas reservoirs has been well established despite the use of biocides and the harsh physicochemical conditions of produced water including high salinity (greater than 35,000 mg/L) and pressure (5,000 to 12,000 psi), variable temperatures (around 100°C), and low oxygen (less than 1%) (Elshahed and Struchtemeyer, 2012; Chen et al., 2014; Daly et al., 2016; Lipus et al., 2018; Gulliver et al., 2021; Stemple et al., 2021). Biological activity is generally disadvantageous in hydraulic fracturing operations as certain microbial metabolisms can contribute to bacterial hydrogen sulfide and acid production, leading to reservoir souring and microbial-influenced corrosion (MIC). Additionally, the formation of biofilms can result in biofouling, characterized by the undesirable accumulation of microorganisms, and bioclogging, where microbial cells accumulate within the pores of materials potentially damaging infrastructure and obstructing pipelines (Bakke et al., 1992; Youssef et al., 2009; Gieg et al., 2011; Gaspar et al., 2014). These detrimental microbial processes can greatly impact the efficiency and recovery of hydrocarbons, cause subsequent infrastructure damage, decrease economic benefits, and exacerbate the environmental impacts of extraction operations (Gaspar et al., 2014). It is necessary to understand the metabolic potential of microorganism in shale gas environments to predict and limit the influence of undesirable biological activity in energy extraction operations of shale gas reservoirs.

There is limited knowledge on what occurs in shale gas reservoirs during the initial production phase once the shut-in period process has concluded. Prior work investigating the microbial ecology of shale gas produced water has established a general trend in microbial community structure that reveals a shift in microbial composition from diverse populations introduced through fluid injection to a more heterogenous community often dominated by thermo- and halotolerant anaerobic microbes (Mohan et al., 2013; Murali Mohan et al., 2013; Mouser et al., 2016). Previous studies have shown *Halanaerobium* to be a pervasive genus throughout the production phase and the dominant community member in produced water examined from both the Marcellus and Bakken Shales (Lipus et al., 2017, 2018; Tinker et al., 2022). This genus is capable of fermentation and thiosulfate reduction (Daly et al., 2016; Liang et al., 2016; Lipus et al., 2017). Other halo- and thermotolerant members of Bacillota and Proteobacterial taxa including *Pseudomonas*, *Acinetobacter*, *Halomonas*, *Marinobacter*, and archaeal groups including *Methanohalophilus* and *Methanosarcina* are prevalent shale taxa associated with production fluid microbiomes from more characterized shale plays (Davis et al., 2012; Mohan et al., 2013; Cluff et al., 2014; Tucker et al., 2015; Daly et al., 2016).

Several of the studies referenced above have used 16S rRNA gene analysis to classify important microbial communities associated with hydraulic fracturing production from a variety of shale formations including the Marcellus, Bakken, Antrim, and Barnett shales (Davis et al., 2012; Cluff et al., 2014; Mohan et al., 2014; Tucker et al., 2015; Lipus et al., 2018; Stemple et al., 2021). Although 16S rRNA studies provide insight into the microbial community and structure of these environments, this technique can also be fundamentally limited in its ability to understand the functional capacity and metabolic linkages within these crucial systems. Additional information is needed to understand the functional potential of key taxa identified including metagenomic surveys generated from whole genome sequencing. There has been impressive, yet a limited number of metagenomic studies examining shale-produced fluids to evaluate the functional potential of microbial communities, limited almost entirely to the Marcellus Shale (Mohan et al., 2014; Daly et al., 2016; Booker et al., 2017; Lipus et al., 2017; Booker et al., 2019). Daly et al. (2016) reconstructed genomes from produced water collected from hydraulically fractured Marcellus and Utica Shales to investigate the functional roles of abundant halotolerant taxa observed along with metabolite analysis of the produced water. This study has led to subsequent laboratory studies investigating dominant *Halanaerobium* strains isolated from sampled Utica formation fluids, which have revealed the metabolic potential for subsurface biofilm formation and biogenic sulfide production catalyzed by the *Halanaerobium* bacteria (Booker et al., 2017, 2019). Lipus et al. (2017) also investigated Marcellus Shale wells, reconstructing and annotating a *Halanaerobium* draft genome that showed genetic evidence for fermentation, thiosulfate reduction, and biofilm formation pathways. These studies highlight the necessity of broadening our understanding of microbial metabolism within hydraulically fractured oil and gas wells across the major United States shale plays. They also underscore the potential for detrimental processes, such as microbial-induced corrosion and sulfide production, which can have adverse impacts on the infrastructure, operations, and recovery efficiency of the oil and gas industry.

The objective of this study was to assess the composition, abundance, and functional potential of microbial communities in production fluid collected in the first 35 days of production following a three-week shut-in period in the Delaware Basin. This assessment was also evaluated and compared with the pre-shut-in fracture fluid and unreacted proppant material. We sought to understand how the microbial community structure would evolve throughout early phase production of the Hydraulic Fracture Test Site 2 (HFTS 2) located in one of the most prolific oil and gas plays in the United States and examine the microbial functional potential of this production fluid. Furthermore, we aimed to characterize the metabolic profile of an abundant *Clostridia* species observed to be highly enriched throughout most of the early phase production in our initial 16S rRNA analysis. This work can be used to understand important biological activity that occurs in the early production phase of hydraulic fracturing and contribute to the growing knowledge of produced water microbiology that can help mitigate undesirable events such as MIC, well souring, and biofouling in hydraulic fracturing operations.

## Materials and methods

### Sampling and DNA isolation

Sampling and DNA isolation were performed by collaborators at the Gas Technology Institute (GTI). Production fluid was sampled over a 35-day period spanning from August 15, 2019, to September 19, 2019, at the Hydraulic Fracture Test Site 2 (HFTS 2) in the Delaware-Wolfcamp Formation. Fracture fluid and unreacted proppant were also sampled. DNA was extracted from produced fluid collected from the HFTS 2 site following the DNeasy Powersoil kit as well as four kit blanks (QIAGEN, Hilden, Germany).

### Quantitative PCR

The microbial load was measured using previously described quantitative polymerase chain reaction (qPCR) methodology at the National Energy Technology Laboratory (NETL, Pittsburgh, PA) to assess the relative abundance of microbes in produced water samples (Tinker et al., 2020). Briefly, 16S rRNA gene primers (F: GTGSTGCAYGGYTGTCGTCA; R: ACGTCRTCCMCACCTTCCTC) designed by Maeda et al. (2003) were used with an expected amplicon size of 146 base pairs. qPCR reactions were run in triplicate on a Magnetic Induction Cycler (MIC) (Bio Molecular Systems, Upper Coomera, Australia). Each reaction contained 2X SensiFAST SYBR No-Rox master mix (Bioline, London, United Kingdom), 400 nM forward primer, 400 nM reverse primer, and 1 μL of template DNA for a total reaction of 20 μL. Conditions for qPCR consisted of a polymerase activation step at 95°C for 2 min followed by 40 amplification cycles each consisting of: denaturation at 95°C for 5 s, annealing at 62°C for 5 s, and an extension step at 72°C for 1 s. Standard curves were generated using gBlocks Gene Fragments (Integrated DNA Technologies, Coralville, IA, United States) (Supplementary Figure S1), and we included negative control samples in each amplification assay.

### 16S rRNA gene sequencing and analysis

Isolated DNA was amplified using universal primers targeting the V4 region of the 16S gene, as has been described by Caporaso et al. (2011, 2012). Extraction blanks were PCR amplified as well to ensure no contamination had occurred. Samples were visualized using an Agilent Bioanalyzer to assess DNA quality. We confirmed that the bioanalyzer contained a strong peak in the area of interest (approximately 350 bp for the V4 amplicon with adapters and barcodes). If there were visible primer dimers or non-specific PCR amplification on the bioanalyzer, we did an additional round of cleaning and/or repeated the PCR when possible. DNA libraries were then constructed following manufacturer’s instructions and sequenced on an Illumina MiSeq (Illumina, San Diego, CA) using a 300 cycle V2 Nano kit. Raw sequences were deposited to NCBI under BioProject PRJNA1068269.

**Figure 1:**
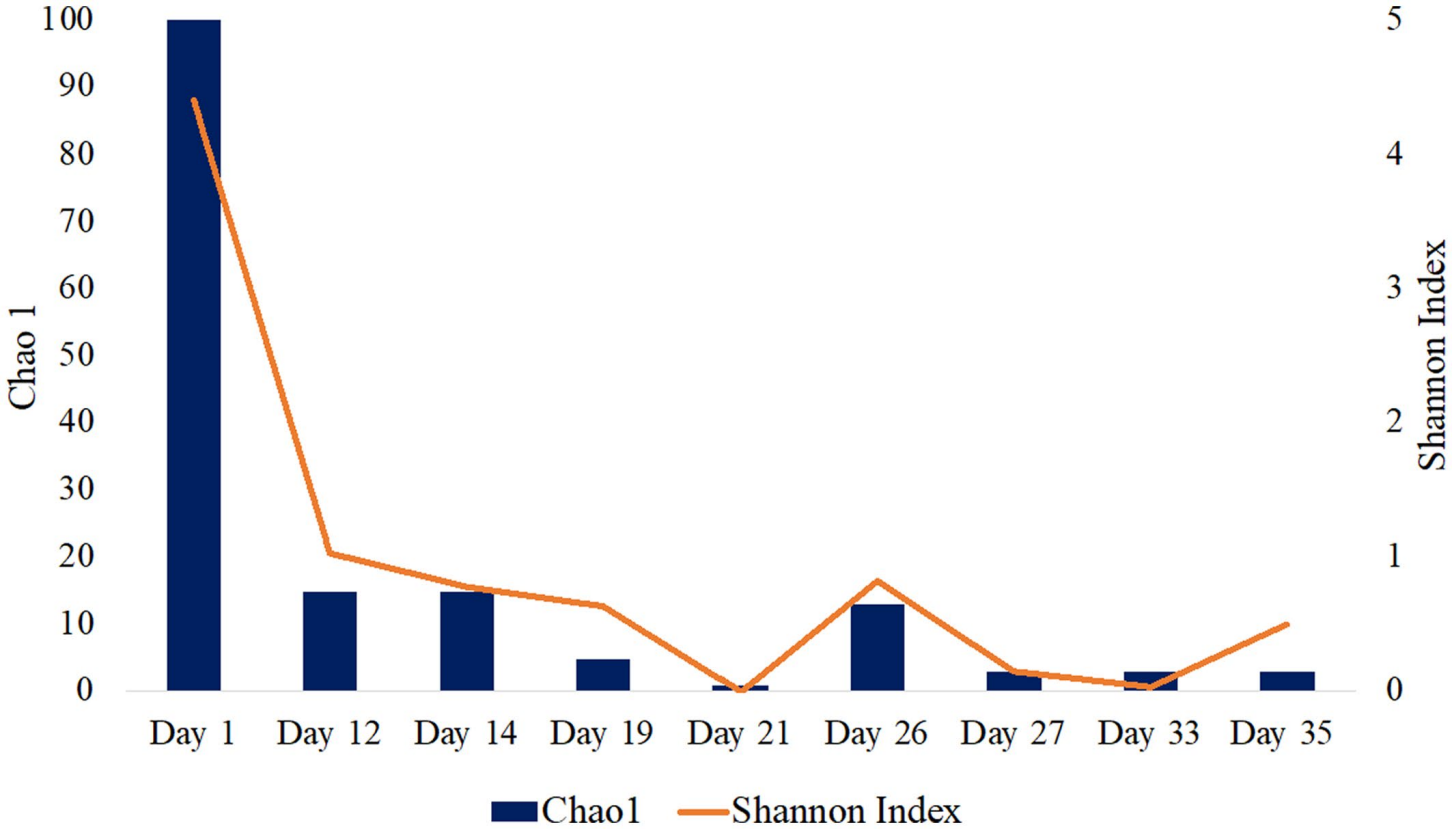
Alpha diversity analysis of fluid production samples during HFTS 2 production. Days 5 and 7 were not included as there was not enough detectable DNA for diversity analysis.

16S rRNA gene single-end sequences were analyzed using Quantitative Insights into Microbial Ecology (QIIME) 2 pipeline version 2021.20 (Bolyen et al., 2019). Sequences were imported using EMPSingleEndSequences and demultiplexed using demux emp-single command. Sequences were then denoised and quality trimmed using the DADA2 denoising software package wrapped in QIIME2, with default settings and a truncation length of 250 bps. The Q2-diversity plugin was used to analyze alpha diversity metrics including Chao1 and Shannon Indexes (Figure 1; Table 1). The classify-sklearn (Pedregosa et al., 2011) command was used in order to classify taxonomy of the representative sequences using a pre-trained Naïve Bayes classifier trained on Silva 132 99% OTUs from the 515F/806R region (Quast et al., 2013; Yilmaz et al., 2014). Initial 16S rRNA sequence processing and analysis have been previously described in Gulliver et al. (2021) presented at the Unconventional Resources Technology Conference.

**Table 1:** Alpha diversity results for Wolfcamp formation production fluid determined by 16S rRNA sequencing.

Sample day	Number of Reads	Richness^R^	Diversity^D^	16S rRNA gene copies/mL sample
Day 1	6,277	100	4.40	3.83×10^4^
Day 5	BDL	BDL	BDL	BDL
Day 7	BDL	BDL	BDL	BDL
Day 12	8,613	15	1.03	1.37×10^4^
Day 14	4,937	15	0.78	6.81×10^3^
Day 19	5,307	5	0.64	6.06×10^5^
Day 21	312	1	N/A	6.73×10^6^
Day 26	8,028	13	0.83	2.60×10^4^
Day 27	1,671	3	0.15	2.13×10^6^
Day 33	2,106	3	0.03	9.08×10^7^
Day 35	3,957	3	0.50	1.67×10^6^
Frac Fluid	1,932	29	3.60	4.96×10^6^
Unreacted Proppant	1,877	52	4.91	3.24×10^5^

^R^Chao1 Index, ^D^Shannon Index.

### Metagenomic sequencing

Isolated DNA from Day 33 was selected for shotgun metagenomic sequencing to further investigate the metabolic potential of the highly enriched *Clostridia* species identified to be almost 100% abundant in the Day 33 16S rRNA gene data. Isolated DNA was sequenced at the SeqCenter (formerly, Microbial Genome Sequencing Center) (Pittsburgh, Pennsylvania), and whole shotgun metagenomic sequencing was performed at a depth of 1 Gpb on the NextSeq 2000 platform following the Illumina manufacturer’s instructions available at support.illuminia.com. The resultant reads were paired-end reads (2×151) delivered as FASTQ files. Shotgun metagenome sequencing generated 22,301,506 total paired-end reads. Raw sequences were deposited to NCBI under BioProject PRJNA1068269.

### Metagenomic assembly, binning, and analysis

Quality of total sequenced DNA was assessed using FASTQC v0.11.9, and paired-end reads were trimmed with Trimmomatic v0.36. Reads were then assembled using IDBA-UD v1.1.13 with default parameters (Peng et al., 2012). The minimum contig length was 500 bp, the maximum length was 60,262 bp, and the N50 was 10,786 bp. Additional assembly information is included in Supplementary Table S1. Read recruitment back to assemblies was performed via BowTie2 v2.3.2, and metagenomic assemblies were binned with MetaBAT2 v1.7 to recover metagenome-assembled genomes (MAGs) (Langmead and Salzberg, 2012; Kang et al., 2015). Bins were quality assessed using QUAST v4.4, CheckM was used to assign a percent completion and contamination for each metagenomic bin, and three high-quality bins (>90% completion, <5% contamination) were recovered from our Day 33 production fluid sample (Table 2; Gurevich et al., 2013; Parks et al., 2015). All three MAGs were taxonomically classified using GTDB-Tk v1.0.2 (Chaumeil et al., 2020). Metagenomic assemblies were then annotated using DRAM v0.1.0 using default parameters (Shaffer et al., 2020).

**Table 2:** Metagenome characteristics and taxonomic assignments of 3 MAGs isolated from HFTS 2 production fluid.

	Bin name	MAG 1	MAG 2	MAG 3
Sequence statistics	Genome size	1,353,951	2,429,042	1,832,813
	Number of contigs	137	226	166
	tRNA count	25	42	29
	N50	12,854	14,934	15,012
	GC content (%)	32.87	33.49	34.38
	Completion (%)	85.5	89.0	90.0
	Contamination (%)	0.48	0.35	1.75
Taxonomic assignments	Domain	Archaea	Bacteria	Bacteria
	Phylum	Methanobacteriota	Bacillota	Deferribacterota
	Class	Methanococci	Clostridia	Deferribacteres
	Order	Methanococcales	Peptostreptococcales	Deferribacterales
	Family	*Methanococcaceae*	*Caminicellaceae*	*Flexistipitaceae*
	Genus	*Methanothermococcus*	*Petromonas*	–
	Species	*M. thermolithotrophicus*	*P. tenebris*	–

### Metabolic profiling of HFTS 2 MAGs

DRAM annotations were used to make inferences about the important metabolic pathways of major players in this subsurface system, including the heavily enriched *Clostridia* species. Gene annotations were confirmed using RASTtk v1.073 and Kyoto Encyclopedia of Genes and Genomes (KEGG) to identify the presence or absence of specific genomic features (protein encoding genes and RNA) involved in microbial metabolisms including fermentation pathways, sulfur metabolism, biofilm production, and methanogenesis (Brettin et al., 2015; Kanehisa et al., 2021). These pathways were chosen because the biological production of acid, sulfide, and biofilms can lead to biocorrosion and biofouling that can cause structural and functional damage within the hydraulic fracturing infrastructure (Caporaso et al., 2011; Kahrilas et al., 2015).

## Results

### Microbial abundance of production fluid

The microbial biomass load of the Wolfcamp Formation region was determined via qPCR. The microbial loads for the production fluid samples ranged from 10^1^ to 10^5^ 16S rRNA gene copies/mL (Table 1). The fracture fluid (frac fluid) used during injection for the hydraulic fracturing process and unreacted proppant were analyzed as well. Both frac fluid and unreacted proppant showed abundances of 5.0×10^6^ and 3.4×10^5^ 16S rRNA gene copies/mL sample, respectively, indicating a considerable microbial population injected into the well. Our analysis of frac fluid and unreacted proppant revealed a relatively high microbial population size, comparable to the levels observed in the later weeks of production. There was not enough detectable DNA to allow for abundance analysis at Day 5 or 7, suggesting a substantial decrease in the microbial population occurred after the first day of production. However, we did observe an increase in biomass load starting on Day 12 of production, with the highest biomass from the produced water load being detected on Day 33.

### Microbial diversity and 16S rRNA community composition of early production fluid

The microbial diversity of the collected samples was analyzed by calculating the richness and diversity within each sample based on the ASV table obtained by 16S rRNA amplicon sequencing (Figure 1). Our analysis of frac fluid and proppant showed a relatively high diversity compared with most production fluid samples, with Chao1 indexes of 29 and 52 and Shannon Indexes of 3.60 and 4.91 for the frac fluid and unreacted proppant, respectively (Table 1). This suggests that the fluids injected into the well had a relatively diverse microbial population. Chao1 index ranged between 1 and 100 and the Shannon diversity index ranged between 0.03 and 4.40 for all samples, with Day 1 having the most diverse microbial community among fluid production samples.

**Figure 2:**
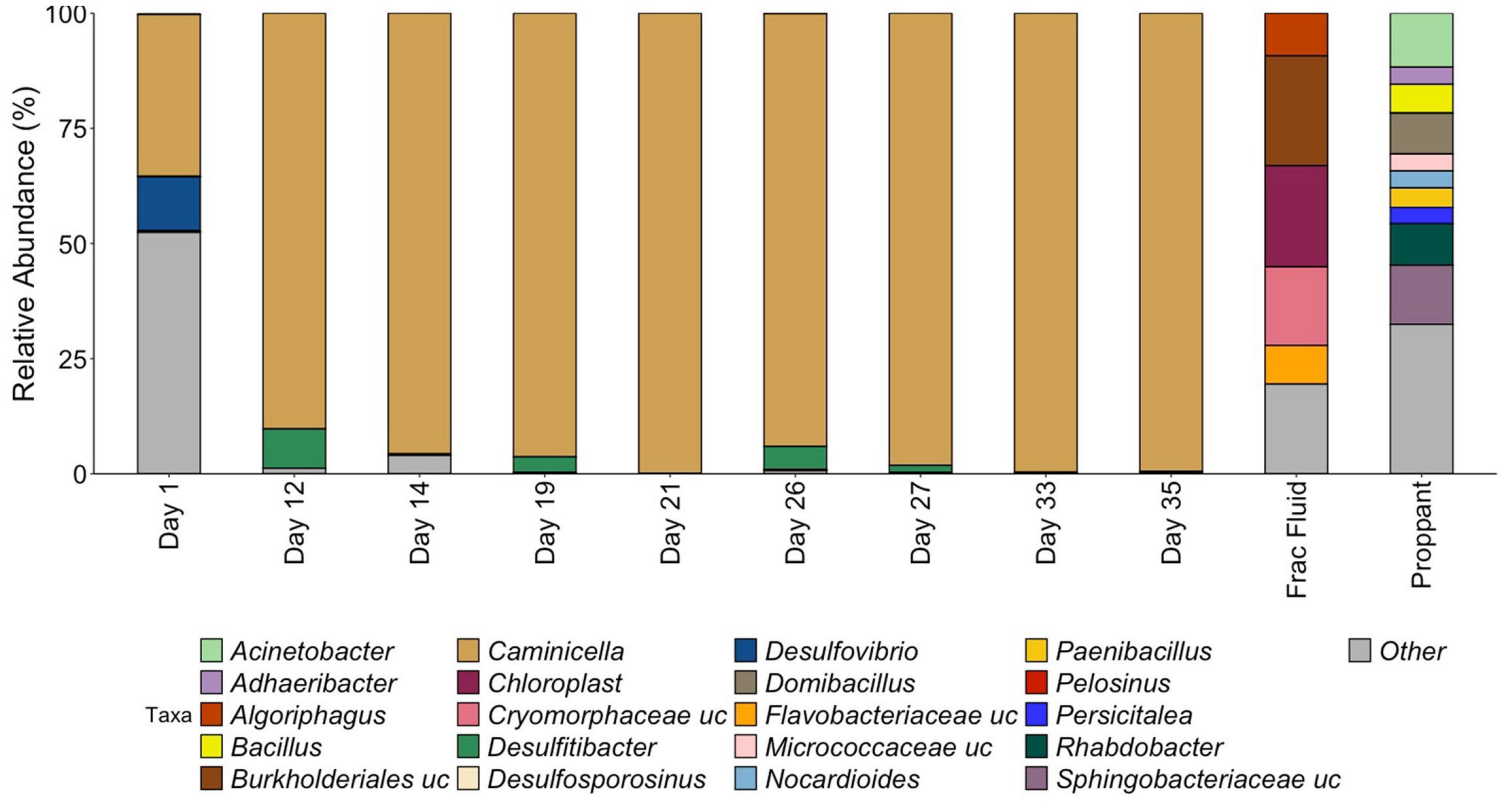
Relative abundance of samples during HFTS 2 production. All relative abundance is listed at the genus level, with the exception of uncultured family level groups that could not be further resolved. All listed genera are above 3% relative abundance, with the remaining genus grouped in “Other.” “.uc” represents uncultured groups.

The microbial community composition was first assessed by 16S rRNA gene sequencing (Figure 2). The starting frac fluid and unreacted proppant had a higher diversity consisting of several environmental bacterial groups with a wide variety of metabolisms that have been classified from various environments including ground water and mud (Alegado et al., 2013; Tatar, 2018; Bowman, 2020). Post shut-in, microbial diversity was relatively high for the first day of production. The Day 1 microbial community was predominantly comprised of numerous low-abundance microorganisms, with only two genera, *Caminicella* (35%) and *Desulfovibrio* (12%), representing more than 10% of the community. This was reflected by the high diversity (Shannon Index 4.40, Chao1 Index 100) for Day 1, which was greater than all other days observed, including the initial frac fluid and unreacted proppant. The observed rebound in microbial load after 12 days of production was accompanied by a sharp decrease in diversity with the emergence of *Caminicella*, a single taxon that dominated the system. *Caminicella*, initially undetected in both the frac fluid and the unreacted proppant, exhibited a 35% relative abundance on the first day of production. By Day 12, this genus was highly enriched in the system, surpassing 90%; appearing to outcompete the more biodiverse, less abundant populations that were initially observed.

### Metagenome-assembled genomes provide metabolic insights significant to natural gas recovery

Three metagenome-assembled genomes (MAGs), described in Table 2, were obtained from our metagenomic analysis of Day 33 HFTS 2 production fluid. All three MAGs belong to taxonomic groups that have been previously detected in subsurface unconventional oil and gas reservoirs (Pannekens et al., 2019; Christman et al., 2020; Tinker et al., 2020; Scheffer et al., 2021). Although our 16S rRNA sequencing of Day 33 production fluid demonstrated a relative abundance of 99% of a *Clostridia* species closely related to *Caminicella* sp., two additional high-quality MAGs were recovered from shotgun metagenomic sequencing. Whole genome metagenomic analysis putatively resolved the unknown *Clostridia* species as the thermophilic *Petromonas tenebris,* part of the divergent clostridial lineage isolated from a high salt, high temperature oil reservoir (Christman et al., 2020). The smallest genome was identified as *Methanothermococcus thermolithotrophicus,* a thermophilic, hydrogenotrophic methanogen that has been previously identified in produced waters recovered from the Permian Basin in Texas (Tinker et al., 2020). Our final MAG was resolved to the Family level, classified as a member of the *Flexistipitaceae* family. Together the 16S rRNA and metagenomic findings provide a putative framework for the *in situ* microbiome of the HFTS 2 gas reservoir during early phase production.

Ecosystem-relevant putative metabolisms that were identified in the three recovered MAGs are summarized in Supplementary Figure S3, and detailed annotations of proteins of interests are provided in the subsequent section (Supplementary Figure S3). The following discussion explores the metabolic potential of our annotated MAGs and presents genetic evidence pertaining to sulfur, mixed acid fermentation, and methanogenesis pathways that are critical to the hydraulic fracturing industry.

### Sulfur cycling

Microbial-induced corrosion (MIC) has been previously connected to the presence of sulfate and thiosulfate-reducing activity in microorganisms, leading to the production of hydrogen sulfide (Liang et al., 2016). Sulfide production in the well can cause biogenic souring, which can consequently instigate operational, environmental, and recovery deficiencies. Wells that contain more than 4 ppmv of H_2_S are considered “sour,” and the occurrence of sour gas can be toxic and dangerous for operation workers as well as lead to pitting of steel and stress corrosion of that can damage structural materials such as metal pipes compromising well infrastructure (Choudhary et al., 2015; Gaspar et al., 2016). This led us to search for genes involved in both sulfate and thiosulfate sulfidogenesis to better characterize the sulfur respiration potential in our recovered MAGs.

Annotation of our metagenomic sequences did not uncover the presence of any dissimilatory sulfate reductase genes (*dsrAB*) involved in classical sulfate reduction. Our search did identify genes for thiosulfate reduction in both the *P. tenebris* and *Flexistipitaceae* draft genomes. This included thiosulfate sulfurtransferase (TST)/rhodanese genes and genes coding for anaerobic sulfite reductase asrABC (*AsrA*, *AsrB*, *AsrC*), involved in sulfite reduction, which reduces sulfite to sulfide under anaerobic conditions (Hallenbeck et al., 1989). This analysis also revealed several protein encoding genes including two rhodanese genes, *rdlA*, a thiosulfate sulfurtransferase involved in anaerobic thiosulfate reduction of thiosulfate to sulfite and *Mpst* that converts thiosulfate to adenylyl sulfate (Ravot et al., 2005). These genes have previously been described in *Halanaerobium* sp., including *H. congolense*, *H. saccharolyticum*, and *H. T82-1*, a moderately halophilic bacterium known to reduce thiosulfate and sulfur into sulfide, as well as other unidentified anaerobic, thiosulfate-reducing *Clostridiales* such as SRL 4198 (Ravot et al., 1995, 2005; Daly et al., 2016). We identified all three anaerobic sulfite reductase genes, *AsrA*, *AsrB*, and *AsrC,* in the *P. tenebris* draft genome. We also observed the presence of a thiosulfate reductase/polysulfide reductase in the *Flexistipitaceae* draft genome, indicating this species may also perform dissimilatory thiosulfate reduction as part of its sulfur metabolism.

### Mixed acid fermentation

Our MAG annotation analysis also revealed the presence of genes associated with mixed acid fermentation, specifically indicated by the presence of genes related to short-chain fatty acids (SCFA) and alcohol conversions provided by DRAM. Identification of these genes indicates putative metabolic pathways that could contribute to acid production in produced water leading to MIC. We discovered several genes in the *P. tenebris* draft genome involved in pyruvate metabolisms, including *ldh,* which encodes a lactate dehydrogenase involved in the conversion of pyruvate to lactate; *adh*, which encodes for an alcohol dehydrogenase that converts simple sugars to ethanol; and two genes involved in the *Pfl* complex that encode for pyruvate formate-lyase that transforms pyruvate to hydrogen and carbon dioxide (Table 3; Knappe and Sawers, 1990; Farhana and Lappin, 2024). Furthermore, acetate kinase, *ack*, which facilitates the reversible reaction of acetyl-phosphate to acetate, was identified in both *P. tenebris* and *Flexistipitaceae* MAGs, *and Pta,* which encodes a phosphate acetyltransferase that converts Acetyl-CoA to acetate, was found in *Flexistipitaceae,* but not in the *P. tenebris* (Table 3; Boynton et al., 1996). The Pta-Ack pathway is critical for the anaerobic production of acetate and ATP production. This pathway has been identified in many bacteria, for which under anaerobic growth *Pta* catalyzes the conversion of acetyl-CoA to acetyl-phosphate that is then subsequently transformed to acetate by *Ack* coupled with the production of ATP (Kim et al., 2015). Given the existence of both these genes in the *Flexistipitaceae* MAG, it suggests potential acetate metabolism by the Pta-Ack pathway. Genes responsible for the conversation of pyruvate to hydrogen and carbon dioxide were also identified including Pyruvate formate-lyase (*Pfl*) and Pyruvate formate-lyase activating enzyme (*Pfl-ae*) (Table 3). The identification of genes involved in mixed acid fermentation, including SCFA and alcohol conversions, sheds light on the putative metabolisms in the production fluid microbiome. The potential conversion of pyruvate into fermentation products (lactate, acetate, ethanol, hydrogen, and carbon dioxide) strongly suggests that the key players in this subsurface reservoir may play a role in the production of acid in the HFTS 2 natural gas reservoir.

**Table 3:** Proteins of interest for hydraulic fracturing industry identified in MAG annotation.

Thiosulfate reduction			
Protein	EC number	Putative function	MAG
Thiosulfate sulfurtransferase, Rhodanese **Mpst**	2.8.1.1	Thiosulfate to adenylyl sulfate	*P. tenebris*
Rhodanese-like gene **RdlA**	N/A	Adenylyl sulfate to sulfite	*P. tenebris*
Thiosulfate reductase ***PhsA***/polysulfide reductase ***PsrA***	EC 1.8.5.5	Dissimilatory reduction of thiosulfate	*Flexistipitaceae*
Assimilatory sulfite reductase (ferredoxin)	E.C. 1.8.7.1	Sulfate assimilation	*P. tenebris*
Anaerobic sulfite reductases **AsrA**	N/A	Sulfite reduction	*P. tenebris*
Anaerobic sulfite reductases **AsrB**	N/A	Sulfite reduction	*P. tenebris*
Anaerobic sulfite reductases **AsrC**	EC 1.8.1-	Sulfite reduction	*P. tenebris*

Mixed acid fermentation			
Protein	EC number	Putative function	MAG
Lactate dehydrogenase **Ldh**	EC 1.1.1.27	Pyruvate to lactate	*P. tenebris*
Lactic acid dehydrogenase (cytochrome)	EC 1.1.2.3	Pyruvate metabolism	*P. tenebris*
Lactate dehydrogenase complex protein **LldF**	N/A	Carbohydrate metabolism	*Flexistipitaceae*
Phosphate acetyltransferase **Pta**	EC 2.3.1.8	Acetyl-CoA to acetyl-phosphate	*Flexistipitaceae*
Acetate Kinase **Ack**	EC 2.7.2.1	Acetyl-phosphate to acetate	*P. tenebris, Flexistipitaceae*
Propionate CoA-transferase	EC 2.8.3.1	Acetyl-CoA to acetate	*P. tenebris*
Alcohol dehydrogenase **Adh**	EC 1.1.1.1	Pyruvate to ethanol	*P. tenebris*
Pyruvate formate-lyase **Pfl**	EC 2.3.1.54	Pyruvate to hydrogen and carbon dioxide	*P. tenebris*
Pyruvate formate-lyase activating enzyme **Pfl-ae**	EC 1.97.1.4	*Pfl* complex	*P. tenebris, Flexistipitaceae*

Methanogenesis			
Protein	EC number	Putative function	MAG
Methyl-coenzyme M reductase system component A2 Mcr-Component A	N/A	Methane Metabolism	*M. thermolithotrophicus*
N^5^-methyl-tetrahydromethanopterin-coenzyme M methyltransferase **Mtr**	EC 2.1.1.86	Coenzyme M to Methyl-coenzyme M	*M. thermolithotrophicus*
Formylmethanofuran dehydrogenases **Fwd**	EC 1.2.99.5	N-formylmethanofuran to CO_2_	*M. thermolithotrophicus*

Biofilm formation			
Protein	EC number	Putative function	MAG
RNA-binding protein **SpoVG**	N/A	Biofilm activator	*P. tenebris*
Sporulation two-component response regulator **Spo0A**	N/A	Biofilm activator	*P. tenebris*
Polysaccharide biosynthesis proteins **PelA-G**	N/A	Polysaccharide biosynthesis	*Flexistipitaceae*
Glycosyltransferase, family 2	2.4.183	Cellulose synthesis	*M. thermolithotrophicus*
Glycosyltransferase group 2 family protein gene **Glt2**	N/A	Capsular-polysaccharide synthesis	*P. tenebris*
GGDEF domain protein	N/A	Cellulose production	*P. tenebris, Flexistipitaceae*
Diguanylate cyclase gene **AdrA**	N/A	Cellulose biosynthesis induction	*P. tenebris, Flexistipitaceae*

Specific protein indicated in bold.

### Methanogenesis

Methanogenesis functional genes were investigated in our *Methanothermococcus thermolithotrophicus* draft genome, including the methyl-coenzyme M reductase system (MCR components), essential in anaerobic microbial methane metabolism (Shima et al., 2012). This organism is classified as a moderately thermophilic, hydrogenotrophic methanogenic archaea known to reduce carbon dioxide to methane and has been previously detected in high temperature, saline reservoirs (Nikolova and Gutierrez, 2022). We identified methyl-coenzyme M reductase (MCR) Component A, a protein involved in the final step of methane production in methanogenesis (Bonacker et al., 1993; Kuhner et al., 1993). We also discovered several genes that encode for conserved enzymes in hydrogenotrophic methanogenesis. These include N^5^-methyl-tetrahydromethanopterin-coenzyme M methyltransferase (MTR) that transfers the methyl group to coenzyme M, which is then subsequently reduced to methane as well as formylmethanofuran dehydrogenases, *Fwd*, which catalyzes the reaction of formylmethanofuran to carbon dioxide (Bertram et al., 1994; Upadhyay et al., 2016; Berghuis et al., 2019; Zhang et al., 2020). It is important to note that these proteins can also be found in methanogens capable of acetoclastic and methylotrophic methanogenesis. However, while *Methanothermococcus thermolithotrophicus* is a hydrogenotrophic methanogen, categorizing this MAG as hydrogenotrophic based solely on the presence of these three genes may not be conclusive. These findings do suggest, however, the potential of biogenic methane production in the evaluated production fluid.

### Biofilm formation

The presence of biofilm formation genes was also examined in the MAGs. Biofilm formation in engineered subsurface systems has been associated with accelerated MIC in well systems and clogging of fractures, resulting in infrastructure damage and decreased gas recovery (Elshahed and Struchtemeyer, 2012; Vengosh et al., 2014). We identified SpoVG and Spo0A genes in our *P. tenebris* draft genome, which have been studied in *B. subtilis* and known to be involved in sporulation and biofilm activation (Stanley and Lazazzera, 2004). Specifically, SpoVG is located upstream of Spo0A and appears to participate in biofilm formation by regulating transcription of *spo0A*, a gene involved in surface attachment initiation for biofilm formation (Huang et al., 2021). GGDEF domain protein, which includes Diguanylate cyclase (*adrA*), involved in the biosynthesis and production of cellulose, and glycosyltransferase group 2 family protein gene (*glt2*), involved in polysaccharide synthesis, has been associated with the production of exopolysaccharide (EPS) that provides structural support for biofilm formation and was found in both bacterial MAGs (Stanley and Lazazzera, 2004; Whiteley and Lee, 2015). Polysaccharide biosynthesis proteins, PelA-G, were first identified in *Pseudomonas aeruginosa*, a pathogenic bacterium that serves as a model system for biofilm development (Ryder et al., 2007). This seven-gene operon is involved in the production of extracellular matrix, including polysaccharide biosynthesis and maintenance of the biofilm structure, and all seven genes were identified in the *Flexistipitaceae* genome annotation.

## Discussion

This study examined the microbiome of both pre-shut-in frac fluid and production fluid samples obtained within the initial 35 days of production of Hydraulic Fracture Test Site 2 (HFTS 2) in the Wolfcamp Formation of the Delaware Basin. We aimed to analyze the microbial community during hydraulic fracturing production following a shut-in period of 3 weeks, compare it to pre-shut-in material, and evaluate the metabolic potential of the subsurface hydraulic fracturing microbiome. Furthermore, this study provides additional understanding of the microbial community dynamics and potential metabolic processes occurring in the subsurface throughout the early stages of production in one of the most prolific oil and gas regions in the United States. 16S rRNA analysis provided a taxonomic classification of production fluid during different days of production, followed by a metagenomic analysis of Day 33 production fluid that enabled insights into the metabolic potential of this engineered ecosystem with important implications for hydrocarbon recovery from the deep biosphere.

### Clostridia species dominate Wolfcamp formation production fluid

16S rRNA analysis revealed that during the initial days of production of HFTS 2, there was a significant shift in community composition that did not reflect the microbial ecology of the fracture fluid or unreacted proppant. By Day 12, the microbial community had become highly enriched for a *Clostridia* species in the Bacillota phylum. This, coupled with a strong decrease in diversity, demonstrates a distinct selection for this species within the engineered system. 16S rRNA taxonomic classification most closely identified this species a member of the *Caminicella* clade within the thermophilic *Clostridiales* order, which dominated through the subsequent days of sampling.

Little is known about *Caminicella* except that it has been previously isolated from a Pacific Rise hydrothermal vent and is a thermophilic, heterotrophic anaerobe (Alain et al., 2002). Clostridial species are known to persist in hydrocarbon systems due to their ability to survive at high temperatures and salinity and have been implicated as potential reservoir souring culprits given the existence of sulfate reduction pathways identified in *Clostridiales* genomes isolated from hydrocarbon reservoirs (Youssef et al., 2009; Kobayashi et al., 2012; Silva et al., 2013; Hu et al., 2016; Vigneron et al., 2017; Kim et al., 2018). Members of *Clostridiales* are abundant in produced waters collected from shale regions, including the Marcellus, Antrim, and Bakken Shale (Lipus et al., 2017; Tinker et al., 2020; Stemple et al., 2021). This group of bacteria are obligate anaerobic organisms that use fermentation pathways and sulfur metabolism and are spore forming; however, the role of this clostridial species in reservoir microbiology has not been well characterized (Christman et al., 2020).

Given the high abundance and population density of this enriched taxon and its previous implication in biocorrosion and well souring, we chose to further classify the functional potential of the *Clostridia* microorganism in this system using metagenomic analysis. We assembled a near-complete clostridial draft genome (89%) that most closely identified as a member of the recently classified *Petromonas tenebris* lineage. This novel clostridial group was recently identified by Christman et al. (2020), a study which found nearly 25% of the genes present in their *P. tenebris* bins represented the *Caminicella* genus (Tatar, 2018).

### Functional potential of isolated MAGs from production fluid

Understanding the microbial processes and metabolic capabilities of microorganisms in oil and gas reservoirs can help predict detrimental biological processes, including MIC, souring, or biofilm-mediated fouling. These processes are often a consequence of microbial metabolisms that result in acid, sulfide, and biofilm production that may affect the efficacy and efficiency of oil and gas recovery (Youssef et al., 2009; Gregory et al., 2011). Metagenomic profiling of our Day 33 production fluid allowed for the assembly of three draft genomes containing genes with putative functions for thiosulfate reduction, acid production, methane generation, and biofilm formation.

Metagenomic profiling of our recovered *P. tenebris* draft genome revealed metabolic pathways capable of contributing to microbial sulfide production in the subsurface. This includes the presence of genes involved in sulfite reduction, which can produce sulfide in oxygen-depleted environments. We also observed the metabolic potential for thiosulfate reduction catalyzed by rhodanese enzymes encoded by thiosulfate sulfurtransferase genes. The presence of rhodanese and asr-encoding genes has been well established in *Clostridia*, and several studies have shown the potential to convert thiosulfate to sulfide (Vengosh et al., 2014; Upadhyay et al., 2016). *RdlA* and *Mpst* genes have been described in thiosulfate-reducing anaerobes, including *Halanaerobium congolense,* and are putatively responsible for thiosulfate-dependent sulfidogenesis involving the conversion of thiosulfate to adenylyl sulfate to sulfite (Ravot et al., 2005). Thiosulfate reduction encoding genes have been described previously in *Clostridia* shale genomes including *Halanaerobium* MAGs identified in produced water recovered from the Marcellus and Utica Shales and laboratory culture-based approaches using *Halanaerobium* strains obtained from formation water samples collected from these regions (Daly et al., 2016; Lipus et al., 2017; Booker et al., 2019; Cliffe et al., 2020). Furthermore, MAGs of *Petromonas tenebris* linages recovered from produced fluids of a hot oil well were found to possess sulfur metabolism genes, including anaerobic sulfite reductase (ASR) and sulfite reductase (ferredoxin), which were both identified in our *P. tenebris* MAG and are typically part of the assimilatory sulfate reduction pathway (Tatar, 2018). These results provide genetic evidence of thiosulfate reduction by *P. tenebris* and indicate the potential for this abundant species to contribute to microbial sulfide production in this shale reservoir.

High-temperature, high-pressure simulation experiments performed by Gulliver et al. (2021) using fracture fluid, shale, and proppant from HFTS 2 found that the shut-in process increased sulfate concentrations. In this experiment, the sulfate levels in the initial fracturing fluid (approximately 1,605 mg/L) nearly doubled after 7 days of the shut-in simulation, averaging approximately 3,272 mg/L in the duplicate reactors. Mineralogy and fluid chemistry analysis data suggest that elevated sulfate concentrations were likely a result of initial dissolution of sulfate after introduction of the fracture fluid to the reservoir shale matrix followed by precipitation (Gulliver et al., 2021). These findings suggest that dissolution reactions that occur during shut-in could result in increased sulfate concentration during this early stage of production. Elevated levels of sulfate in the reservoir during this initial phase could help explain the immediate shift that was observed using the 16S rRNA analysis at the beginning of production with the presence of sulfate reducing bacteria *Desulfovibrio,* representing 12% of the community on Day 1. As production continued, we observed the strong selection for the *Caminicella* sp. for which the subsequent metagenomic analysis revealed the *P. tenebris* MAG associated with thiosulfate reduction in our Day 33 production fluid. The ability of the *Caminicella* sp. to outperform *Desulfovibrio* and other low abundant taxa present in the initial days of production is evidenced by its near-complete dominance of the microbial community by Day 12. This abundance suggests a greater adaptability of *Caminicella* sp. to the changing environmental conditions present throughout production, including variable sulfate levels as well as high temperature and pressure.

Biofilm formation is used as a tool for microbial life to survive in harsh environments such as shale ecosystems; however, the existence of biofilms in natural gas formations can lead to bioclogging and biofouling and decrease the efficacy of biocides (Bottero et al., 2010; Struchtemeyer et al., 2012; Cluff et al., 2014). Biofilm-forming ability has been associated with several *Clostridia* species, and previous studies investigating unconventional reservoirs have identified *Clostridia* genes encoding proteins involved in biofilm processes. Functional studies by Lipus et al. (2017) and Booker et al. (2019) both investigated the genus *Halanaerobium*, a member of the *Clostridia* class. Lipus et al. (2017) reconstructed and annotated a draft genome from produced water sampled from the Marcellus Shale and identified genes encoding for sporulation processes, surface attachment proteins, and exopolysaccharide (EPS) production, all important processes in biofilm formation. Laboratory studies by Booker et al. (2019) incubated *Clostridia* isolates from a natural gas well in the Utica Point Pleasant formation at representative subsurface pressures and observed cell clumping among Clostridial biomass and increased abundance of proteins involved in the synthesis of EPS. The identification of genes involved in biofilm activation, surface attachment, and EPS biosynthesis found in our *P. tenebris* draft genome reveals the functional capacity of *Petromonas* sp. to form biofilms in hydrocarbon environments. These findings align with previous studies on Clostridial functions in shale ecosystems and offer evidence of biofilm-forming abilities within this genus, which have not been previously characterized (Ðapa et al., 2012; Charlebois et al., 2017; Lipus et al., 2017).

Biological methane production is also an important process in shale gas reservoirs. Several studies have investigated the prevalence and classification of methanogens in shale gas reservoirs, including the well-characterized Marcellus Shale and the Barnett and Antrim Shales, to better understand biogenic methane production and methanogenic activity in subsurface shale environments (Tucker et al., 2015; Lipus et al., 2016; Borton et al., 2018). Microbial gas formation in the subsurface is responsible for biogenic gas shales, and association of methanogenic archaea with fermentative organisms has been shown to enhance MIC (Wuchter et al., 2013; Liang et al., 2014). MAGs recovered from produced fluid collected from the Permian Basin identified a near-complete *Methanothermococcus thermolithotrophicus* draft genome. Although the genus *Methanohalophilus* has been shown to be a very prevalent methanogen in several studies investigating methanogenic archaea in hydraulically fractured shales gas reservoir, *Methanothermococcus* MAGs have also been recovered from microbial community studies of the Midland Basin and found to be a dominant microbial community member in oil production wells sampled in north-central Louisiana (Shelton et al., 2016; Borton et al., 2018; Tinker et al., 2022). *Methanothermococcus* species can grow using a wide variety of sulfur sources, including sulfide, elemental sulfur, thiosulfate, sulfite, sulfate, and nitrogen sources, including ammonium, nitrate, and N_2_ gas. This species of archaea has also been identified in high-temperature, saline, anaerobic environments, including marine oil reservoir waters and sediments (Whitman, 2015). The production of sulfide and fermentation products by our bacterial MAG could serve as potential substrates for *M. thermolithotrophicus* growth in the subsurface. Draft genome analysis of this species also revealed the presence of genes involved in methane metabolism including a component of MCR, a central enzyme in methanogenesis (Chen et al., 2020). *M. thermolithotrophicus* is a hydrogenotrophic methanogen. Therefore, the identification of these genes is unsurprising; however, it does illuminate aspects of the energy metabolism of this methanogenic archaea, conveying the putative function of this species to reduce one carbon substrate to methane via *Mtr* protein complex (Upadhyay et al., 2016). Together, these findings elucidate the potential anaerobic microbial community structure in the production fluid of the Delaware Basin and the potential implications for the oil and gas industry in this region.

### Study implications and conclusion

Hydraulic fracturing is the most common practice for extraction of natural gas from shale formations. The United States Geological Survey (USGS) estimated that the Wolfcamp and overlying Bone Spring formations are likely the largest oil and gas resources ever to be assessed in the United States with the Delaware-Wolfcamp play housing over 220 trillion cubic feet of natural gas (U.S. Energy Information Administration, 2022). Microbial activity within these shale operations can have major consequences on energy recovery in this highly exploited region; therefore, greater understanding of the potential metabolic process that will occur in these reservoirs and production fluids is necessary to limit the occurrence of microbial-influenced corrosion, biofouling, and well souring issues.

In this study, we assessed the microbiological changes that occurred over the first 35 days of production from fluid samples collected from a horizontally drilled hydraulic fracturing well in the Wolfcamp formation. We found a highly enriched *Clostridia* species that appeared to outcompete all other subsurface species present in the reservoir within the first few days of production. This finding aligns with previously established observations of low diversity, microbial populations that persists within the same shale play (Lipus et al., 2017, 2018; Tinker et al., 2020).

This study also evaluated the metabolic potential of the Day 33 production fluid sample recovering three near-complete MAGs including a *Clostridia* draft genome that was most closely related to the newly classified *Petromonas tenebris* and is the most likely representative of the predominant *Clostridia* species observed in our 16S rRNA profile. Two of our three annotated draft genomes, including the *P. tenebris* and *Flexistipitaceae* MAGs, revealed the genetic potential for thiosulfate reduction, fermentation pathways, and biofilm formation. These discoveries suggest that these species could contribute to sulfide production within the reservoir and production fluid and may play a role in biofilm formation. Our third MAG was classified as a thermophilic methanogen with genetic evidence of methane production and the potential to use byproducts of fermentation as substrates for growth.

In conclusion, this study sheds light on the production fluid microbiome of one of the most prolific shale plays in the United States, showing the enrichment of a thermophilic, fermentative anaerobe with the potential for sulfide production and biofilm formation with important implications for hydraulic fracturing operations, energy recovery, and environmental impact of produced water. This study contributes to the growing research area investigating the metabolic potential of microorganisms in hydrocarbon resource recovery operations from unconventional natural gas reservoirs and expands the knowledge of microbial ecology in the Delaware Basin production fluid.

## Data availability statement

The datasets presented in this study can be found in online repositories. The names of the repository/repositories and accession number(s) can be found in the article/Supplementary material.

## Author contributions

BS: Conceptualization, Data curation, Formal analysis, Investigation, Methodology, Writing – original draft. DG: Conceptualization, Formal analysis, Methodology, Project administration, Supervision, Writing – review & editing. PS: Investigation, Writing – review & editing. KT: Investigation, Writing – review & editing. KB: Conceptualization, Investigation, Supervision, Writing – review & editing.

## References

[ref1] Alain K. Pignet P. Zbinden M. Quillevere M. Duchiron F. Donval J.-P.. (2002). *Caminicella sporogenes* gen. nov., sp. nov., a novel thermophilic spore-forming bacterium isolated from an East-Pacific rise hydrothermal vent. Int. J. Syst. Evol. Microbiol. 52, 1621–1628. doi: 10.1099/00207713-52-5-1621, PMID: 12361265

[ref2] Alegado R. A. Grabenstatter J. D. Zuzow R. Morris A. Huang S. Y. Summons R. E.. (2013). Algoriphagus machipongonensis sp. nov., co-isolated with a colonial choanoflagellate. Int. J. Syst. Evol. Microbiol. 63, 163–168. doi: 10.1099/ijs.0.038646-0, PMID: 22368173 PMC3709532

[ref3] Bakke R. Rivedal B. Mehan S. (1992). Oil reservoir biofouling control. Biofouling 6, 53–60. doi: 10.1080/08927019209386209

[ref4] Berghuis B. A. Yu F. B. Schulz F. Blainey P. C. Woyke T. Quake S. R. (2019). Hydrogenotrophic methanogenesis in archaeal phylum Verstraetearchaeota reveals the shared ancestry of all methanogens. Proc. Natl. Acad. Sci. 116, 5037–5044. doi: 10.1073/pnas.1815631116, PMID: 30814220 PMC6421429

[ref5] Bertram P. A. Karrasch M. Schmitz R. A. Böcher R. Albracht S. P. Thauer R. K. (1994). Formylmethanofuran dehydrogenases from methanogenic Archaea. Substrate specificity, EPR properties and reversible inactivation by cyanide of the molybdenum or tungsten iron-sulfur proteins. Eur. J. Biochem. 220, 477–484. doi: 10.1111/j.1432-1033.1994.tb18646.x 8125106

[ref6] Bolyen E. Rideout J. R. Dillon M. R. Bokulich N. A. Abnet C. C. Al-Ghalith G. A.. (2019). Reproducible, interactive, scalable and extensible microbiome data science using QIIME 2. Nat. Biotechnol. 37, 852–857. doi: 10.1038/s41587-019-0209-9, PMID: 31341288 PMC7015180

[ref7] Bonacker L. G. Baudner S. Mörschel E. Böcher R. Thauer R. K. (1993). Properties of the two isoenzymes of methyl-coenzyme M reductase in Methanobacterium thermoautotrophicum. Eur. J. Biochem. 217, 587–595. doi: 10.1111/j.1432-1033.1993.tb18281.x, PMID: 8223602

[ref8] Booker A. E. Borton M. A. Daly R. A. Welch S. A. Nicora C. D. Hoyt D. W.. (2017). Sulfide generation by dominant Halanaerobium microorganisms in hydraulically fractured shales. mSphere 2:e00257-17. doi: 10.1128/mSphereDirect.00257-17, PMID: 28685163 PMC5497025

[ref9] Booker A. E. Hoyt D. W. Meulia T. Eder E. Nicora C. D. Purvine S. O.. (2019). Deep-subsurface pressure stimulates metabolic plasticity in shale-colonizing Halanaerobium spp. Appl. Environ. Microbiol. 85:e00018-19. doi: 10.1128/AEM.00018-19 30979840 PMC6544827

[ref10] Borton M. A. Daly R. A. O’Banion B. Hoyt D. W. Marcus D. N. Welch S.. (2018). Comparative genomics and physiology of the genus Methanohalophilus, a prevalent methanogen in hydraulically fractured shale. Environ. Microbiol. 20, 4596–4611. doi: 10.1111/1462-2920.14467, PMID: 30394652

[ref11] Bottero S Picioreanu C Enzien M van Loosdrecht MC Bruining H Heimovaara T. (2010). Formation damage and impact on gas flow caused by biofilms growing within proppant packing used in hydraulic fracturing. Paper presented at the SPE International Symposium and Exhibition on Formation Damage Control, Lafayette, Louisiana, USA.

[ref12] Bowman J. P. (2020). Out from the shadows – resolution of the taxonomy of the family Cryomorphaceae. Front. Microbiol. 11:795. doi: 10.3389/fmicb.2020.00795, PMID: 32431677 PMC7214798

[ref13] Boynton Z. L. Bennett G. N. Rudolph F. B. (1996). Cloning, sequencing, and expression of genes encoding phosphotransacetylase and acetate kinase from *Clostridium acetobutylicum* ATCC 824. Appl. Environ. Microbiol. 62, 2758–2766. doi: 10.1128/aem.62.8.2758-2766.1996, PMID: 8702268 PMC168061

[ref14] Brettin T. Davis J. J. Disz T. Edwards R. A. Gerdes S. Olsen G. J.. (2015). RASTtk: a modular and extensible implementation of the RAST algorithm for building custom annotation pipelines and annotating batches of genomes. Sci. Rep. 5:8365. doi: 10.1038/srep08365, PMID: 25666585 PMC4322359

[ref15] Caporaso J. G. Lauber C. L. Walters W. A. Berg-Lyons D. Huntley J. Fierer N.. (2012). Ultra-high-throughput microbial community analysis on the Illumina HiSeq and MiSeq platforms. ISME J. 6, 1621–1624. doi: 10.1038/ismej.2012.8, PMID: 22402401 PMC3400413

[ref16] Caporaso J. G. Lauber C. L. Walters W. A. Berg-Lyons D. Lozupone C. A. Turnbaugh P. J.. (2011). Global patterns of 16S rRNA diversity at a depth of millions of sequences per sample. Proc. Natl. Acad. Sci. 108, 4516–4522. doi: 10.1073/pnas.1000080107, PMID: 20534432 PMC3063599

[ref17] Charlebois A. Jacques M. Boulianne M. Archambault M. (2017). Tolerance of *Clostridium perfringens* biofilms to disinfectants commonly used in the food industry. Food Microbiol. 62, 32–38. doi: 10.1016/j.fm.2016.09.009, PMID: 27889162

[ref18] Chaumeil P.-A. Mussig A. J. Hugenholtz P. Parks D. H. (2020). GTDB-Tk: a toolkit to classify genomes with the genome taxonomy database. Bioinformatics 36, 1925–1927. doi: 10.1093/bioinformatics/btz848, PMID: 31730192 PMC7703759

[ref19] Chen J. Al-Wadei M. H. Kennedy R. C. M. Terry P. D. (2014). Hydraulic fracturing: paving the way for a sustainable future? J. Environ. Public Health 2014:656824, 1–10. doi: 10.1155/2014/656824 24790614 PMC3984842

[ref20] Chen H. Gan Q. Fan C. (2020). Methyl-coenzyme M reductase and its post-translational modifications. Front. Microbiol. 11:578356. doi: 10.3389/fmicb.2020.578356 33162960 PMC7581889

[ref21] Choudhary L. Macdonald D. Alfantazi A. (2015). Role of thiosulfate in the corrosion of steels: a review. Corrosion 71, 1147–1168. doi: 10.5006/1709

[ref22] Christman G. D. León-Zayas R. I. Zhao R. Summers Z. M. Biddle J. F. (2020). Novel clostridial lineages recovered from metagenomes of a hot oil reservoir. 1. Sci. Rep. 10:8048. doi: 10.1038/s41598-020-64904-6, PMID: 32415178 PMC7229112

[ref23] Cliffe L. Nixon S. L. Daly R. A. Eden B. Taylor K. G. Boothman C.. (2020). Identification of persistent Sulfidogenic Bacteria in shale gas produced waters. Front. Microbiol. 11:286. doi: 10.3389/fmicb.2020.00286, PMID: 32153553 PMC7046593

[ref24] Cluff M. A. Hartsock A. MacRae J. D. Carter K. Mouser P. J. (2014). Temporal changes in microbial ecology and geochemistry in produced water from hydraulically fractured Marcellus shale gas Wells. Environ. Sci. Technol. 48, 6508–6517. doi: 10.1021/es501173p, PMID: 24803059

[ref25] Daly R. A. Borton M. A. Wilkins M. J. Hoyt D. W. Kountz D. J. Wolfe R. A.. (2016). Microbial metabolisms in a 2.5-km-deep ecosystem created by hydraulic fracturing in shales. Nat. Microbiol. 1:16146. doi: 10.1038/nmicrobiol.2016.146 27595198

[ref26] Ðapa T. Leuzzi R. Ng Y. K. Baban S. T. Adamo R. Kuehne S. A.. (2012). Multiple factors modulate biofilm formation by the anaerobic pathogen *Clostridium difficile*. J. Bacteriol. 195, 545–555. doi: 10.1128/JB.01980-12 23175653 PMC3554014

[ref27] Davis J. P. Struchtemeyer C. G. Elshahed M. S. (2012). Bacterial communities associated with production facilities of two newly drilled thermogenic natural gas Wells in the Barnett shale (Texas, USA). Microb. Ecol. 64, 942–954. doi: 10.1007/s00248-012-0073-3, PMID: 22622766

[ref28] Elshahed M. S. Struchtemeyer C. G. (2012). Bacterial communities associated with hydraulic fracturing fluids in thermogenic natural gas wells in north Central Texas, USA. FEMS Microbiol. Ecol. 81, 13–35. doi: 10.1111/j.1574-6941.2011.01196.x 22066833

[ref29] Farhana A. Lappin S. L. (2024). Biochemistry, Lactate DehydrogenaseStatPearls. Treasure Island, FL: StatPearls Publishing. 32491468

[ref30] Gaspar J. Davis D. Camacho C. Alvarez P. J. J. (2016). Biogenic versus thermogenic H2S source determination in Bakken Wells: considerations for biocide application. Environ. Sci. Technol. Lett. 3, 127–132. doi: 10.1021/acs.estlett.6b00075

[ref31] Gaspar J. Mathieu J. Yang Y. Tomson R. Leyris J. D. Gregory K. B.. (2014). Microbial dynamics and control in shale gas production. Environ. Sci. Technol. Lett. 1, 465–473. doi: 10.1021/ez5003242

[ref32] Gieg L. M. Jack T. R. Foght J. M. (2011). Biological souring and mitigation in oil reservoirs. Appl. Microbiol. Biotechnol. 92, 263–282. doi: 10.1007/s00253-011-3542-6, PMID: 21858492

[ref33] Gregory K. B. Vidic R. D. Dzombak D. A. (2011). Water management challenges associated with the production of shale gas by hydraulic fracturing. Elements 7, 181–186. doi: 10.2113/gselements.7.3.181

[ref34] Gulliver D. Sarkar P. Tinker K. Means N. Fazio J. Xiong W.. (2021). “Novel geochemistry determined from high pressure” in High temperature simulation experiments of hydraulic fracture test site 2 (One Petro). Paper presented at the SPE/AAPG/SEG Unconventional Resources Technology Conference, Houston, Texas, USA.

[ref35] Gurevich A. Saveliev V. Vyahhi N. Tesler G. (2013). QUAST: quality assessment tool for genome assemblies. Bioinformatics 29, 1072–1075. doi: 10.1093/bioinformatics/btt086, PMID: 23422339 PMC3624806

[ref36] Hallenbeck P. C. Clark M. A. Barrett E. L. (1989). Characterization of anaerobic sulfite reduction by salmonella typhimurium and purification of the anaerobically induced sulfite reductase. J. Bacteriol. 171, 3008–3015. doi: 10.1128/jb.171.6.3008-3015.1989, PMID: 2656637 PMC210008

[ref37] Hu P. Tom L. Singh A. Thomas B. C. Baker B. J. Piceno Y. M.. (2016). Genome-resolved metagenomic analysis reveals roles for candidate Phyla and other microbial community members in biogeochemical transformations in oil reservoirs. mBio. doi: 10.1128/mBio.01669-15 PMC4725000 26787827

[ref38] Huang Q. Zhang Z. Liu Q. Liu F. Liu Y. Zhang J.. (2021). SpoVG is an important regulator of sporulation and affects biofilm formation by regulating Spo0A transcription in *Bacillus cereus* 0–9. BMC Microbiol. 21:172. doi: 10.1186/s12866-021-02239-6, PMID: 34102998 PMC8186074

[ref39] Kahrilas G. A. Blotevogel J. Stewart P. S. Borch T. (2015). Biocides in hydraulic fracturing fluids: a critical review of their usage, mobility, degradation, and toxicity. Environ. Sci. Technol. 49, 16–32. doi: 10.1021/es503724k, PMID: 25427278

[ref40] Kanehisa M. Furumichi M. Sato Y. Ishiguro-Watanabe M. Tanabe M. (2021). KEGG: integrating viruses and cellular organisms. Nucleic Acids Res. 49, D545–D551. doi: 10.1093/nar/gkaa970, PMID: 33125081 PMC7779016

[ref41] Kang D. D. Froula J. Egan R. Wang Z. (2015). MetaBAT, an efficient tool for accurately reconstructing single genomes from complex microbial communities. PeerJ 3:e1165. doi: 10.7717/peerj.1165, PMID: 26336640 PMC4556158

[ref42] Kim J. N. Ahn S.-J. Burne R. A. (2015). Genetics and physiology of acetate metabolism by the Pta-Ack pathway of *Streptococcus mutans*. Appl. Environ. Microbiol. 81, 5015–5025. doi: 10.1128/AEM.01160-15, PMID: 25979891 PMC4495203

[ref43] Kim D. D. O’Farrell C. Toth C. R. A. Montoya O. Gieg L. M. Kwon T.. (2018). Microbial community analyses of produced waters from high-temperature oil reservoirs reveal unexpected similarity between geographically distant oil reservoirs. Microb. Biotechnol. 11, 788–796. doi: 10.1111/1751-7915.13281, PMID: 29806176 PMC6011920

[ref44] Knappe J. Sawers G. (1990). A radical-chemical route to acetyl-CoA: the anaerobically induced pyruvate formate-lyase system of *Escherichia coli*. FEMS Microbiol. Rev. 6, 383–398. doi: 10.1111/j.1574-6968.1990.tb04108.x, PMID: 2248795

[ref45] Kobayashi H. Endo K. Sakata S. Mayumi D. Kawaguchi H. Ikarashi M.. (2012). Phylogenetic diversity of microbial communities associated with the crude-oil, large-insoluble-particle and formation-water components of the reservoir fluid from a non-flooded high-temperature petroleum reservoir. J. Biosci. Bioeng. 113, 204–210. doi: 10.1016/j.jbiosc.2011.09.015, PMID: 22019404

[ref46] Kuhner C. H. Lindenbach B. D. Wolfe R. S. (1993). Component A2 of methylcoenzyme M reductase system from Methanobacterium thermoautotrophicum delta H: nucleotide sequence and functional expression by *Escherichia coli*. J. Bacteriol. 175, 3195–3203. doi: 10.1128/jb.175.10.3195-3203.1993, PMID: 8491734 PMC204644

[ref47] Langmead B. Salzberg S. L. (2012). Fast gapped-read alignment with bowtie 2.4. Nat. Methods 9, 357–359. doi: 10.1038/nmeth.1923, PMID: 22388286 PMC3322381

[ref48] Liang R. Davidova I. A. Marks C. R. Stamps B. W. Harriman B. H. Stevenson B. S.. (2016). Metabolic capability of a predominant Halanaerobium sp. in hydraulically fractured gas Wells and its implication in pipeline corrosion. Front. Microbiol. 7:988. doi: 10.3389/fmicb.2016.00988 27446028 PMC4916785

[ref49] Liang R. Grizzle R. Duncan K. McInerney M. Suflita J. (2014). Roles of thermophilic thiosulfate-reducing bacteria and methanogenic archaea in the biocorrosion of oil pipelines. Front. Microbiol. 5:89. doi: 10.3389/fmicb.2014.00089, PMID: 24639674 PMC3944610

[ref50] Lipus D. Roy D. Khan E. Ross D. Vikram A. Gulliver D.. (2018). Microbial communities in Bakken region produced water. FEMS Microbiol. Lett. 365:365. doi: 10.1093/femsle/fny107 29688457

[ref51] Lipus D. Vikram A. Ross D. Bain D. Gulliver D. Hammack R.. (2017). Predominance and metabolic potential of Halanaerobium spp. in produced water from hydraulically fractured Marcellus shale Wells. Appl. Environ. Microbiol. 83:e02659-16. doi: 10.1128/AEM.02659-16 28159795 PMC5377500

[ref52] Lipus D. Vikram A. Ross D. E. Bibby K. (2016). Draft genome sequence of *Methanohalophilus mahii* strain DAL1 reconstructed from a hydraulic fracturing-produced water metagenome. Genome Announc. 4:e00899-16. doi: 10.1128/genomea.00899-16, PMID: 27587817 PMC5009974

[ref53] Maeda H. Fujimoto C. Haruki Y. Maeda T. Kokeguchi S. Petelin M.. (2003). Quantitative real-time PCR using TaqMan and SYBR green for *Actinobacillus actinomycetemcomitans*, *Porphyromonas gingivalis*, *Prevotella intermedia*, tetQ gene and total bacteria. FEMS Immunol. Med. Microbiol. 39, 81–86. doi: 10.1016/S0928-8244(03)00224-4, PMID: 14557000

[ref54] Mohan A. M. Bibby K. J. Lipus D. Hammack R. W. Gregory K. B. (2014). The functional potential of microbial communities in hydraulic fracturing source water and produced water from natural gas extraction characterized by metagenomic sequencing. PLoS One 9:e107682. doi: 10.1371/journal.pone.0107682, PMID: 25338024 PMC4206270

[ref55] Mohan A. M. Hartsock A. Bibby K. J. Hammack R. W. Vidic R. D. Gregory K. B. (2013). Microbial community changes in hydraulic fracturing fluids and produced water from shale gas extraction. Environ. Sci. Technol. 47, 13141–13150. doi: 10.1021/es402928b, PMID: 24088205

[ref56] Mouser P. J. Borton M. Darrah T. H. Hartsock A. Wrighton K. C. (2016). Hydraulic fracturing offers view of microbial life in the deep terrestrial subsurface. FEMS Microbiol. Ecol. 92:fiw166. doi: 10.1093/femsec/fiw166, PMID: 27507739

[ref57] Murali Mohan A. Hartsock A. Hammack R. W. Vidic R. D. Gregory K. B. (2013). Microbial communities in flowback water impoundments from hydraulic fracturing for recovery of shale gas. FEMS Microbiol. Ecol. 86, 567–580. doi: 10.1111/1574-6941.12183, PMID: 23875618

[ref59] Nikolova C. Gutierrez T. (2022). Marine hydrocarbon-degrading bacteria: their role and application in oil-spill response and enhanced oil recovery. In Microbial biodegradation and bioremediation: techniques and case studies for environmental pollution. eds. Das S. Dash H. R.. 2nd ed. (Elsevier) pp. 591–600.

[ref60] Osselin F. Saad S. Nightingale M. Hearn G. Desaulty A.-M. Gaucher E. C.. (2019). Geochemical and sulfate isotopic evolution of flowback and produced waters reveals water-rock interactions following hydraulic fracturing of a tight hydrocarbon reservoir. Sci. Total Environ. 687, 1389–1400. doi: 10.1016/j.scitotenv.2019.07.066, PMID: 31412472

[ref61] Pannekens M. Kroll L. Müller H. Mbow F. T. Meckenstock R. U. (2019). Oil reservoirs, an exceptional habitat for microorganisms. New Biotechnol. 49, 1–9. doi: 10.1016/j.nbt.2018.11.006, PMID: 30502541 PMC6323355

[ref62] Parks D. H. Imelfort M. Skennerton C. T. Hugenholtz P. Tyson G. W. (2015). CheckM: assessing the quality of microbial genomes recovered from isolates, single cells, and metagenomes. Genome Res. 25, 1043–1055. doi: 10.1101/gr.186072.114, PMID: 25977477 PMC4484387

[ref63] Pedregosa F. Varoquaux G. Gramfort A. Michel V. Thirion B. Grisel O.. (2011). Scikit-learn: Machine Learning in Python. J. Mach. Learn. Res. 12, 2825–2830.

[ref64] Peng Y. Leung H. C. M. Yiu S. M. Chin F. Y. L. (2012). IDBA-UD: a de novo assembler for single-cell and metagenomic sequencing data with highly uneven depth. Bioinformatics 28, 1420–1428. doi: 10.1093/bioinformatics/bts174, PMID: 22495754

[ref65] Permian Basin Wolfcamp and Bone Spring Shale Plays Geology Review (2019). U.S. Energy Information Administration (EIA). Available at: https://www.eia.gov/maps/pdf/Wolfcamp_BoneSpring_EIA_Report_July2019.pdf.

[ref66] Quast C. Pruesse E. Yilmaz P. Gerken J. Schweer T. Yarza P.. (2013). The SILVA ribosomal RNA gene database project: improved data processing and web-based tools. Nucleic Acids Res. 41, D590–D596. doi: 10.1093/nar/gks1219, PMID: 23193283 PMC3531112

[ref67] Ravot G. Casalot L. Ollivier B. Loison G. Magot M. (2005). rdlA, a new gene encoding a rhodanese-like protein in Halanaerobium congolense and other thiosulfate-reducing anaerobes. Res. Microbiol. 156, 1031–1038. doi: 10.1016/j.resmic.2005.05.009, PMID: 16085393

[ref68] Ravot G. Ollivier B. Magot M. Patel B. Crolet J. Fardeau M.. (1995). Thiosulfate reduction, an important physiological feature shared by members of the order thermotogales. Appl. Environ. Microbiol. 61, 2053–2055. doi: 10.1128/aem.61.5.2053-2055.1995, PMID: 16535035 PMC1388453

[ref69] Ryder C. Byrd M. Wozniak D. J. (2007). Role of polysaccharides in *Pseudomonas aeruginosa* biofilm development. Curr. Opin. Microbiol. 10, 644–648. doi: 10.1016/j.mib.2007.09.010, PMID: 17981495 PMC2176169

[ref70] Scheffer G. Hubert C. R. J. Enning D. R. Lahme S. Mand J. de Rezende J. R. (2021). Metagenomic investigation of a low diversity, high salinity offshore oil reservoir. Microorganisms 9:2266. doi: 10.3390/microorganisms9112266 34835392 PMC8621343

[ref71] Shaffer M. Borton M. A. McGivern B. B. Zayed A. A. La Rosa S. L. Solden L. M.. (2020). DRAM for distilling microbial metabolism to automate the curation of microbiome function. Nucleic Acids Res. 48, 8883–8900. doi: 10.1093/nar/gkaa621, PMID: 32766782 PMC7498326

[ref72] Shelton J. L. Akob D. M. McIntosh J. C. Fierer N. Spear J. R. Warwick P. D.. (2016). Environmental drivers of differences in microbial community structure in crude oil reservoirs across a methanogenic gradient. Front. Microbiol. 7:1535. doi: 10.3389/fmicb.2016.01535, PMID: 27733847 PMC5039232

[ref73] Shima S. Krueger M. Weinert T. Demmer U. Kahnt J. Thauer R. K.. (2012). Structure of a methyl-coenzyme M reductase from Black Sea mats that oxidize methane anaerobically. Nature 481, 98–101. doi: 10.1038/nature10663, PMID: 22121022

[ref74] Short-Term Energy Outlook – U.S. Energy Information Administration (EIA). Available at: https://www.eia.gov/outlooks/steo/report/natgas.php (Accessed January 15, 2024).

[ref75] Silva T. R. Verde L. C. L. Santos Neto E. V. Oliveira V. M. (2013). Diversity analyses of microbial communities in petroleum samples from Brazilian oil fields. Int. Biodeterior. Biodegrad. 81, 57–70. doi: 10.1016/j.ibiod.2012.05.005

[ref76] Stanley N. R. Lazazzera B. A. (2004). Environmental signals and regulatory pathways that influence biofilm formation. Mol. Microbiol. 52, 917–924. doi: 10.1111/j.1365-2958.2004.04036.x, PMID: 15130114

[ref77] Stemple B. Tinker K. Sarkar P. Miller J. Gulliver D. Bibby K. (2021). Biogeochemistry of the Antrim shale natural gas reservoir. ACS Earth Space Chem 5, 1752–1761. doi: 10.1021/acsearthspacechem.1c00087

[ref78] Stringfellow W. T. Domen J. K. Camarillo M. K. Sandelin W. L. Borglin S. (2014). Physical, chemical, and biological characteristics of compounds used in hydraulic fracturing. J. Hazard. Mater. 275, 37–54. doi: 10.1016/j.jhazmat.2014.04.040, PMID: 24853136

[ref79] Struchtemeyer C. G. Morrison M. D. Elshahed M. S. (2012). A critical assessment of the efficacy of biocides used during the hydraulic fracturing process in shale natural gas wells. Int. Biodeterior. Biodegrad. 71, 15–21. doi: 10.1016/j.ibiod.2012.01.013

[ref80] Tatar A. (2018). “Microbial enhanced oil recovery” in Fundamentals of enhanced oil and gas recovery from conventional and unconventional reservoirs. Ed. A. Bahadori. (Gulf Professional Publishing), 291–508.

[ref81] Tinker K. Gardiner J. Lipus D. Sarkar P. Stuckman M. Gulliver D. (2020). Geochemistry and microbiology predict environmental niches with conditions favoring potential microbial activity in the Bakken shale. Front. Microbiol. 11:1781. doi: 10.3389/fmicb.2020.01781, PMID: 32849400 PMC7406717

[ref82] Tinker K. Lipus D. Gardiner J. Stuckman M. Gulliver D. (2022). The microbial community and functional potential in the Midland Basin reveal a community dominated by both thiosulfate and sulfate-reducing microorganisms. Microbiol. Spectr. 10:e00049-22. doi: 10.1128/spectrum.00049-22 35695567 PMC9430316

[ref83] Tinker Kara Lipus Daniel Sarkar Preom Gulliver Djuna. (2020). The geochemistry and microbial ecology of produced waters from three different unconventional oil and gas regions. Unconventional resources technology conference, 20–22 July 2020, Austin, TX, USA.

[ref84] Tucker Y. T. Kotcon J. Mroz T. (2015). Methanogenic Archaea in Marcellus shale: a possible mechanism for enhanced gas recovery in unconventional shale resources. Environ. Sci. Technol. 49, 7048–7055. doi: 10.1021/acs.est.5b00765, PMID: 25924080

[ref85] U.S. Energy Information Administration (EIA). The Wolfcamp play has been key to Permian Basin oil and natural gas production growth – Today in Energy – U.S. Energy Information Administration (EIA). Available at: https://www.eia.gov/todayinenergy/detail.php?id=37532 (Accessed February 1, 2022).

[ref86] Upadhyay V. Ceh K. Tumulka F. Abele R. Hoffmann J. Langer J.. (2016). Molecular characterization of methanogenic N (5)-methyl-tetrahydromethanopterin: coenzyme M methyltransferase. Biochim. Biophys. Acta 1858, 2140–2144. doi: 10.1016/j.bbamem.2016.06.011, PMID: 27342374

[ref87] Vengosh A. Jackson R. B. Warner N. Darrah T. H. Kondash A. (2014). A critical review of the risks to water resources from unconventional shale gas development and hydraulic fracturing in the United States. Environ. Sci. Technol. 48, 8334–8348. doi: 10.1021/es405118y, PMID: 24606408

[ref88] Vigneron A. Alsop E. B. Lomans B. P. Kyrpides N. C. Head I. M. Tsesmetzis N. (2017). Succession in the petroleum reservoir microbiome through an oil field production lifecycle. 9. ISME J. 11, 2141–2154. doi: 10.1038/ismej.2017.78, PMID: 28524866 PMC5563965

[ref89] Whiteley C. G. Lee D.-J. (2015). Bacterial diguanylate cyclases: structure, function and mechanism in exopolysaccharide biofilm development. Biotechnol. Adv. 33, 124–141. doi: 10.1016/j.biotechadv.2014.11.010, PMID: 25499693

[ref90] Whitman W. B. (2015). “Methanothermococcus gen. nov.” in Bergey’s manual of systematics of Archaea and Bacteria (John Wiley & Sons, Ltd), 1–4.

[ref91] Wuchter C. Banning E. Mincer T. Drenzek N. Coolen M. (2013). Microbial diversity and methanogenic activity of Antrim shale formation waters from recently fractured wells. Front. Microbiol. 4:367. doi: 10.3389/fmicb.2013.00367, PMID: 24367357 PMC3853793

[ref92] Yilmaz P. Parfrey L. W. Yarza P. Gerken J. Pruesse E. Quast C.. (2014). The SILVA and “all-species living tree project (LTP)” taxonomic frameworks. Nucleic Acids Res. 42, D643–D648. doi: 10.1093/nar/gkt1209, PMID: 24293649 PMC3965112

[ref93] Youssef N. Elshahed M. S. McInerney M. J. (2009). “Chapter 6 microbial processes in oil fields: culprits, problems, and opportunities” in Advances in applied microbiology (Academic Press), 141–251. 10.1016/S0065-2164(08)00806-X 19203651

[ref94] Zhang C.-J. Pan J. Liu Y. Duan C.-H. Li M. (2020). Genomic and transcriptomic insights into methanogenesis potential of novel methanogens from mangrove sediments. Microbiome 8:94. doi: 10.1186/s40168-020-00876-z, PMID: 32552798 PMC7302380

