# Supplementary material for: Metagenome-assembled genomes provide insight into the metabolic potential during early production of Hydraulic Fracturing Test Site 2 in the Delaware Basin
Source: Front Microbiol. 2024 Jun 12;15:1376536. doi: 10.3389/fmicb.2024.1376536 (PMC11199900; doi:10.3389/fmicb.2024.1376536)
Supplement: Supplementary file 1 [file Data_Sheet_1.pdf]

Supplementary Information: Metagenome-assembled genomes provide insight into the metabolic potential during early production of Hydraulic Fracturing Test Site 2 in the Delaware Basin

Brooke Stemple[1,3], Djuna Gulliver*[2], Preom Sarkar[2,3], Kara Tinker[2,4], Kyle Bibby*[1,3]

[1]Department of Civil and Environmental Engineering and Earth Sciences, University of Notre Dame, Indiana, [2]National Energy Technology Laboratory (NETL), Pittsburgh, Pennsylvania, [3]Oak Ridge Institute for Science and Education, Oak Ridge, Tennessee, [4]Leidos Research Support Team, Pittsburgh, Pennsylvania, [5]GTI Energy, Des Plaines, Illinois.

*Correspondence: Kyle Bibby (kbibby@nd.edu); Djuna Gulliver (djuna.gulliver@netl.doe.gov)

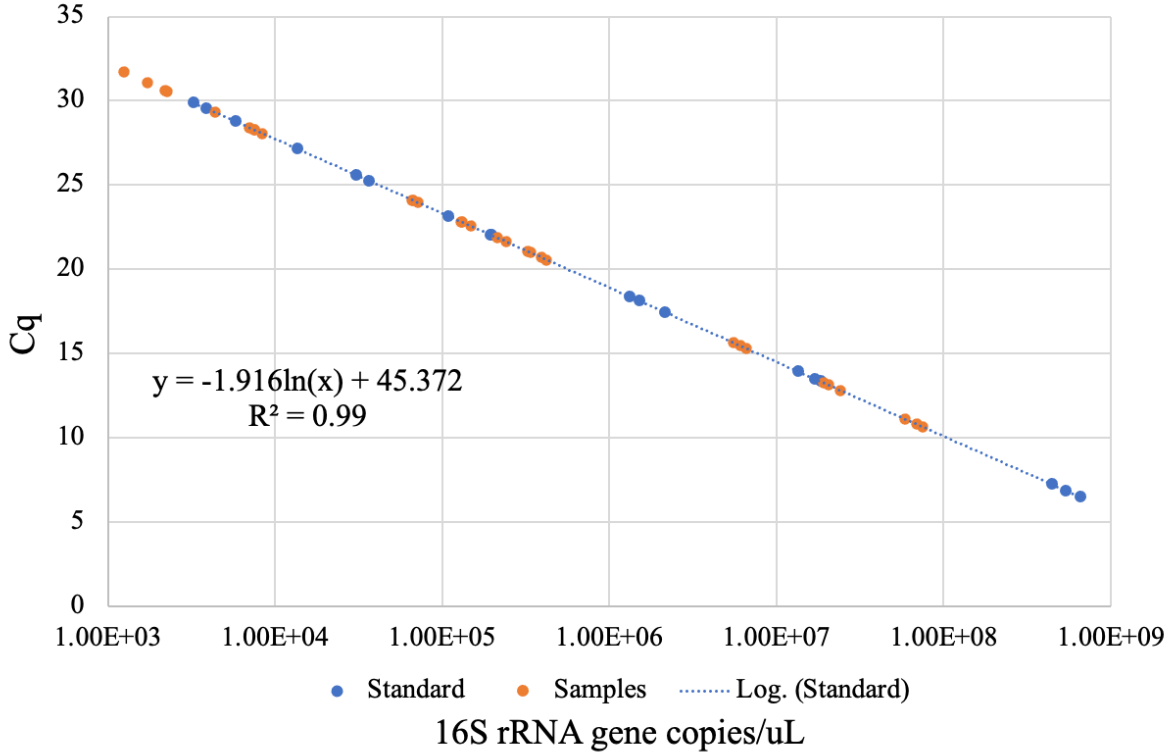

Figure S1 qPCR standard curve. Sample extraction volumes were normalized in order to calculate 16S rRNA gene copies/mL.

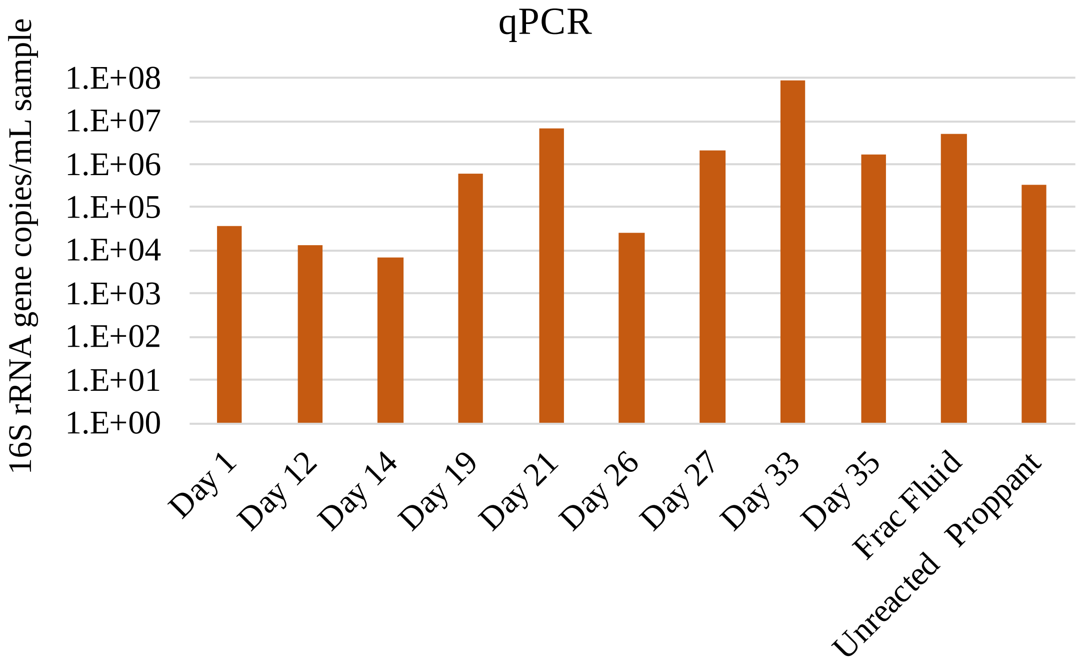

Figure S2. qPCR analysis of sample during HFTS 2 production.

Table S1. Draft genome assembly statistics

**QUAST Assembly Statistics**	**IDBA Assembly**
# of contigs	2,563
Largest contig	60,262
N50	10,786
N75	3,078
GC (%)	34.52
Predicted genes	3,198

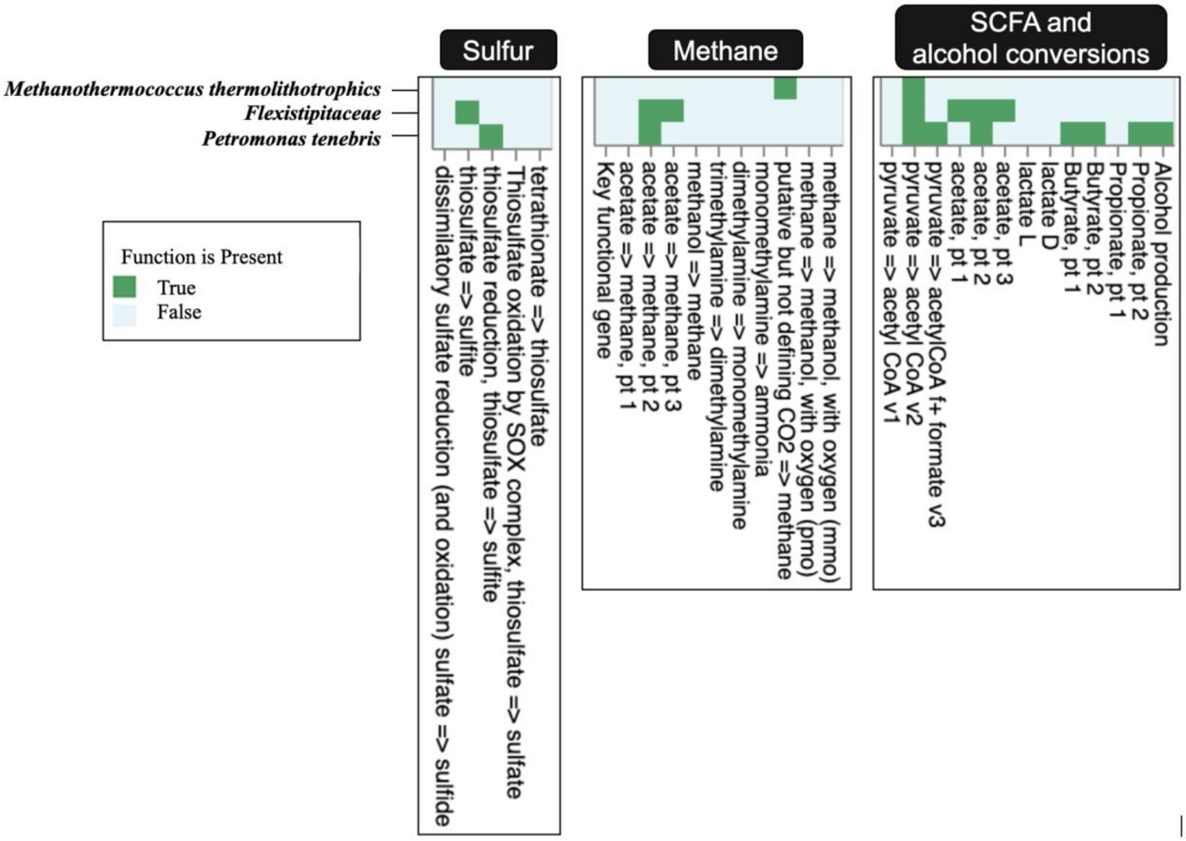

Figure S3. DRAM product visual summary of the ecosystem-relevant metabolic functions of HFTS 2 MAGs. Note: SCFA = short-chain fatty acids.
